# Religious Belief Among Women in Australia: Characteristics and Role in Influencing Children’s Health-Related Quality of Life and Lifestyle

**DOI:** 10.1007/s10943-024-02085-6

**Published:** 2024-07-13

**Authors:** Abbas Bahrampour, Paul Scuffham, Megan Cross, Shu-Kay Ng

**Affiliations:** 1 Department of Biostatistics and Epidemiology, Faculty of Health, Modelling in Health Research Centre, Institute for Futures Studies in Health, Kerman University of Medical Sciences, Kerman, Iran; 2 School of Medicine and Dentistry, Griffith University, Nathan, QLD 4111 Australia; 3 Menzies Health Institute Queensland, Griffith University, Gold Coast, QLD Australia

**Keywords:** Child health, Cohort studies, Maternal health, Quality of life, Religion

## Abstract

Religiosity can be an important factor in women’s health-related behaviour, attitudes, and decision-making. Evidence however, regarding the religiosity of mothers and its influence on child health, is scarce. Based on a large population-based cohort in Australia, we aim to examine the religiosity of women in Australia and the association of maternal religiosity with children’s health-related quality of life (HRQOL) and lifestyle. Our findings indicate that (1) maternal religious involvement was higher for women with higher education levels, ascertained religious values in decision-making, and abstinence from binge drinking in the household, (2) maternal religiosity positively influenced their children’s HRQOL, (3) children of mothers who were more religious had less worries or fewer school-work problems, but the children of mothers with stronger religious beliefs used more internet/computer during the week but had less time playing games on weekends. This study provides additional specificity to inform future health interventions in religious community contexts to enhance the positive influence of maternal religious belief for better development of their children.

## Introduction

In the 2021 Census, 38.9% of Australians reported having no religion (Australian Bureau of Statistics, 2018). Christianity decreased from 86.2% in 1971 to 43.9% in 2021, while other religions increased from 0.8 to 10.0%. Those reporting no religion increased from 6.7 to 38.9% in the same period. Women were more likely than men to report being Christian (55% and 50%, respectively) but the percentages were similar in other religions (8.1% and 8.3%). Further, women (28%) were less likely than men (32%) to have no religion (Australian Bureau of Statistics, 2018).

The religiosity of mothers plays a key role in their health behaviour and attitudes, especially with regard to reproductive health and major decisions (Arousell & Carlbom, 2016). For example, religiosity was found to be positively associated with mothers’ physical and mental health (Piccinini et al., 2021; Seybold & Hill, 2001) and health behaviours (Page et al., 2009). It also influenced their antenatal screening, especially concerning congenital abnormalities and termination (Gitsels-Van Der Wal et al., 2014), their decisions about their children’s education and vaccination (Thomas et al., 2015), and the self-judgement, emotional and motivational development of their children (Bartkowski et al., 2008).

On average, religiosity is associated with a ‘good economic’ attitude, which is defined as being conducive to higher income per capita and growth (Guiso et al., 2003). Religiosity can shape moral and ethical behaviour (Ananthram & Chan, 2016; Barak-Corren & Bazerman, 2017), including attitudes towards the welfare state (Be'ery & Ben-Nun Bloom, 2015), and may influence social behaviour such that individuals with active religiosity are generally more pro-social than non-religious peers (Brañas-Garza et al., 2014; Shariff et al., 2016). The distribution of religiosity changes over time and varies between countries; it can influence people’s decision-making, attitudes towards economic problems, social behaviours and reproductive health care to varying degrees.

To date, there is scant research examining the religiosity of mothers and its association with child health. This study aimed to identify determinants that are associated with mothers’ religiosity and to quantify the relationship of religiosity with decision-making and certain health-related outcomes, such as the health-related quality of life (HRQOL) and lifestyles of their children. The research findings will be valuable to fill the current knowledge gaps about the nature of religiosity in women in Australia and its role in relation to their children’s health-related outcomes. The ultimate goal is to inform future health interventions to enhance the positive influence of maternal religious belief for better development of their children.

## Methods

### Participants

The ‘Environments for Healthy Living’ (EFHL) birth cohort is a population-based study to investigate the relationship between socio-environmental and behavioural factors and the health and development of children in Australia (Cameron et al., 2012; Ng et al., 2016). Women who planned to give birth at one of three participating hospitals were eligible to participate, whereas women younger than 16 years were excluded. Follow-ups via self-report questionnaires occurred when each child turned one, three and five years of age (Cameron et al., 2012). Questions about maternal spirituality were included in the five-year follow-up questionnaires. In this study, we consider 603 mothers with completed religiosity questions.

### Measurements

Religiosity was measured by the Duke University Religion Index (DUREL), which assesses five items in three dimensions of religiosity: one item for organisational religious activity (ORA), one item for non-organisational religious activity (NORA) and three items for intrinsic religiosity (IR) regarding Divine presence and the roles of religious beliefs in approach to life and other dealings in life (Koenig et al., 1997). While ORAs include group activities (e.g., attending religious services or scripture study groups), NORAs are generally performed in private (e.g., prayer). IR reflects the degree of personal commitment to pursuing religion in and of itself or ‘living’ one’s faith (Allport & Ross, 1967; Koenig & Büssing, 2010).

Baseline information was obtained for maternal age, education level and language, and family relationships were measured using the Family Environmental Scale (FES) (Moos & Moos, 1994). Maternal lifestyles included smoking, alcohol and drug use, and private health insurance. The five-year follow-up collected information on marital status, number of children living in the household, household income, household factors (including violence and abuse) and maternal factors concerning the importance of religion in decision-making. Maternal psychological distress was measured using the Kessler-6 (K6) psychological distress scale (Furukawa et al., 2003).

Children’s HRQOL was studied using the Child Health Utility-9D (CHU9D) (Stevens, 2009). The CHU9D aims to explore how young people’s health affects their lives. It has nine dimensions (worried, sad, pain, tired, annoyed, schoolwork, sleep, daily routine and ability to join in activities), each with five increasing levels that illustrate the rank and severity within that dimension (Chen et al., 2015; Stevens, 2010). Additional data on children’s lifestyles included questions about their use of technology (playing game consoles and accessing the internet).

### Statistical Analysis

The data were analysed using Minitab version-17 (Minitab Inc., State College, PA) and IBM SPSS-27 (IBM Corp., NY). The mean and standard deviation were calculated for continuous variables and the frequency by percentage was adopted to describe categorical variables. Ordinal logistic regression was used to identify determinants and children’s HRQOL and lifestyle, associated with religiosity for ORA, NORA and IR, separately. The logit link function was chosen based on likelihood ratio, which provided a better fit for the proportional odds assumption in ordinal regression models.

The goodness-of-fit was assessed using deviance and Pearson chi-square to compare the observed and expected frequencies. The proportional odds assumption was tested using both likelihood ratio and chi-square tests. The significance level was 0.05 for all tests (two-sided).

## Results

### Sample Characteristics

The women had an average age at birth of 30.6 years (range: 16–45 years; Table 1). Most (95.3%) were English-speaking and 3% were bilingual. Regarding marital status, 67.9% were married, 17.5% were living with a partner and 13.5% had separated from their partner in the past 12 months, were single or in a dating relationship. At least 58.5% completed high school (31.4% did not study further, 27.1% completed university degrees) and 28.2% pursued trades. About 55.6% were employed and 36.2% were not in the labour force, and 71.0% of the participants had low psychological distress. Further, 81.1% did not smoke cigarettes but only 42.4% did not consume alcohol during pregnancy. Most participants were satisfied with their living area (90.4%) and felt safe in the community (82.7%).

**Table 1:** Participant characteristics (n = 603)

Characteristics	Frequency (%) or Mean (standard deviation, SD)
Maternal age at birth (years)	30.6 (5.2)
*Spoken language*	
English only Other language only Both Missing	572 (95.3%) 10 (1.7%) 18 (3.0%) 3
*Marital status*	
Married Living with partner Separated, dating or single Divorced/widowed Missing	408 (67.9%) 105 (17.5%) 81 (13.5%) 7 (1.1%) 2
*Maternal education level*	
Did not complete school Completed high school Trade/apprenticeship University degree Missing	80 (13.3%) 189 (31.4%) 170 (28.2%) 163 (27.1%) 1
*Maternal employment*	
Employed Unemployed Not in labour force Missing	325 (55.6%) 48 (8.2%) 212 (36.2%) 18
*Maternal psychological distress*	
Low distress (K6: 0–7) Moderate distress (K6: 8–12) High distress (K6: 13–24) missing	425 (71.0%) 129 (21.6%) 44 (7.4%) 5
*Smoking*	
No Yes Missing	488 (81.1%) 114 (18.9%) 1
*Alcohol intake during pregnancy*	
No Yes Missing	255 (42.4%) 347 (57.6%) 1
*Living area satisfaction*	
Satisfied or very satisfied Neither Dissatisfied or very dissatisfied	545 (90.4%) 38 (6.3%) 20 (3.3%)
*Feel safe in the community*	
No Yes Missing	104 (17.3%) 498 (82.7%) 1
*Eat dinner at the table together*	
0 nights per week 1–4 nights per week ≥ 5 nights per week Missing	58 (9.8%) 224 (37.9%) 309 (52.3%) 12
*Private health insurance*	
No Hospital cover only Extras cover only Both hospital and extras cover Missing	400 (67.0%) 12 (2%) 64 (10.7%) 121 (20.3%) 6
*Household annual income*	
< $60,000 $60,000–$99,999 ≥ $100,000 Missing	221 (38.7%) 180 (31.5%) 170 (29.8%) 32
*Household finance in past year*	
Comfortable and doing alright Just getting by Quite difficult or very difficult Missing	372 (62.2%) 154 (25.8%) 72 (12%) 5
*No. of children* ≤ *16 in the household*	
0 1–3 ≥ 4 missing	268 (45.1%) 313 (52.7%) 13 (2.2%) 9
*Household members consume* ≥ *5 standard drinks on one occasion*	
Never Less than 1 day a week More than 1 day a week	207 (34.3%) 280 (46.4%) 116 (19.3%)
*Religious values in decision making*	
Not important Only a little important Important Missing	222 (37.0%) 106 (17.7%) 272 (45.3%) 3

Regarding household and family characteristics, 90.2% reported eating dinner together at the table at least once a week, with 52.3% doing so five nights or more. While 67% did not have private health insurance, 20.3% had private insurance that covered hospital admission and extras. About 31.5% had an annual income of $60,000–$99,999 and 38.7% earned < $60,000. Whereas 62.2% were comfortable and managing financially, 12% reported their finances as being ‘quite difficult’ or ‘very difficult’ over the past year.

About 45.1% had no children ≤ 16 years in the household and 34.3% had no household member who consumed ≥ 5 standard drinks on one occasion (binge drinking). Regarding religion, 45.3% believed that religious or spiritual values are important in making major decisions but 37% stated that they are not important.

### Organisational Religious Activity (*ORA*)

Table 2 shows that women who attended more ORAs were those who had a higher education level (adjusted odds ratio (AOR) = 1.47; 95% CI: 1.23–1.77; *p* < 0.001), were unemployed (AOR = 2.63 relative to employed; 95% CI: 1.39–5.00; *p* = 0.003), did not smoke during pregnancy (AOR = 2.26; 95% CI: 1.33–3.85; *p* = 0.003), had fewer binge drinking by household members (AOR = 1.14; 95% CI: 1.03–1.26; *p* = 0.012) and considered religion important in decision-making (AOR = 1.39; 95% CI: 1.20–1.59; *p* < 0.001).

**Table 2:** Associations between participant characteristics and five dimensions of religiosity

Characteristics	Adjusted odds ratio (95% CI)				
	ORA	NORA	IR1	IR2	IR3
Maternal education level (higher)	1.47 (1.23, 1.77)	1.46 (1.16, 1.83)	1.32 (1.14, 1.53)	1.39 (1.19, 1.61)	1.37 (1.17, 1.59)
*Maternal employment*					
Employed Unemployed Not in-labour force	Reference 2.63 (1.39, 5.00) 1.40 (0.97, 2.04)				
No smoking during pregnancy	2.26 (1.33, 3.85)	2.11 (1.03, 4.31)			
Living area satisfaction (lower)			1.28 (1.06, 1.55)	1.30 (1.08, 1.58)	
Household annual income (higher quintile)		1.25 (1.08, 1.47)			
Number of children ≤ 16 years living in the household (more)			1.21 (1.05, 1.38)	1.22 (1.06, 1.40)	1.20 (1.04, 1.38)
Household members consume 5 or more standard drinks (less often)	1.14 (1.03, 1.26)	1.21 (1.06, 1.39)	1.13 (1.04, 1.24)	1.17 (1.07, 1.28)	1.18 (1.08, 1.30)
Religious values in decision-making (more important)	1.39 (1.20, 1.59)	1.61 (1.35, 1.92)	1.25 (1.11, 1.43)	1.30 (1.15, 1.47)	1.28 (1.14, 1.45)

### Non-Organisational Religious Activity (NORA)

Table 2 shows that women who engaged in NORAs had higher education levels (AOR = 1.46; 95% CI: 1.16–1.83; *p* = 0.001), did not smoke during pregnancy (AOR = 2.11; 95% CI: 1.03–4.31; *p* = 0.041), had a higher household income (AOR = 1.25; 95% CI: 1.08–1.47; *p* = 0.005), had fewer binge drinking by household members (AOR = 1.21; 95% CI: 1.06–1.39; *p* = 0.007), and considered religion important in decision-making (AOR = 1.61, 95% CI: 1.35–1.92, *p* < 0.001).

### Intrinsic Religious Activity (IRA)

Women who experienced the presence of the Divine were those who had a higher education level (AOR = 1.32; 95% CI: 1.14–1.53; *p* < 0.001), had lower living area satisfaction (AOR = 1.28; 95% CI: 1.06–1.55; *p* = 0.012), had more children ≤ 16 years in the household (AOR = 1.21; 95% CI: 1.05–1.38; *p* = 0.008), had fewer binge drinking by household members (AOR = 1.13; 95% CI: 1.04–1.24; *p* = 0.004), and considered religion important in decision-making (AOR = 1.25; 95% CI: 1.11–1.43; *p* < 0.001).

Women who believed that their religious beliefs determined their approach to life had higher education levels (AOR = 1.39; 95% CI: 1.19–1.61; *p* < 0.001), had lower living area satisfaction (AOR = 1.30; 95% CI: 1.08–1.58; *p* = 0.007), has more children ≤ 16 years in the household (AOR = 1.22; 95% CI: 1.06–1.40; *p* = 0.005), had fewer binge drinking by household members (AOR = 1.17; 95% CI: 1.07–1.28; *p* = 0.001), and considered religion important in decision-making (AOR = 1.30; 95% CI: 1.15–1.47; *p* < 0.001).

Women who tried hard to carry their religion over into all other dealings in life were those who had higher education levels (AOR = 1.37; 95% CI: 1.17–1.59; *p* < 0.001), has more children ≤ 16 years in the household (AOR = 1.20; 95% CI: 1.04–1.38; *p* = 0.011), had fewer binge drinking by household members (AOR = 1.18; 95% CI: 1.08–1.30; *p* < 0.001) and considered religion important in decision-making (AOR = 1.28; 95% CI: 1.14–1.45; *p* < 0.001).

### Children’s Health-Related Quality of Life

From Table 3, the majority reported no problems in the worried (95.8%), sadness (91.1%), pain (93.1%), annoyed (84.3%) and sleep (82.9%) dimensions. However, 24.3% of the children felt a little bit tired and 12.6% reported a few problems in their schoolwork. Further, 17.8% had a few problems in their daily routine, but 74.5% of children could still participate in any activities. The mean CHU9D utility values presented in Table 3 show the general trend of lower HRQOL for children who reported more problems in the nine dimensions (all significant at the 0.05 level).

**Table 3:** Mental health components of participants’ children (CHU9D, n = 603)

Mental health component	Frequency (%)	Mean utility score (SD)
*Worried*		
Does not feel like this Feels a little bit like this Feels a bit like this Feels quite like this Missing	571 (95.8) 19 (3.2) 5 (0.8) 1 (0.2) 7	0.946 (0.06) 0.832 (0.09) 0.786 (0.11) 0.880 (n/a)
*Sadness*		
Does not feel like this Feels a little bit like this Feels a bit like this Missing	543 (91.1) 48 (8.1) 5 (0.8) 7	0.951 (0.05) 0.843 (0.07) 0.800 (0.11)
*Pain*		
Does not feel like this Feels a little bit like this Feels a bit like this Feels quite like this Missing	554 (93.1) 28 (4.7) 10 (1.7) 3 (0.5) 8	0.949 (0.06) 0.855 (0.07) 0.849 (0.07) 0.723 (0.11)
*Tiredness*		
Does not feel like this Feels a little bit like this Feels a bit like this Feels quite like this Feels very like this Missing	373 (62.5) 145 (24.3) 53 (8.9) 20 (3.4) 6 (1.0) 6	0.971 (0.04) 0.901 (0.06) 0.879 (0.07) 0.856 (0.08) 0.852 (0.07)
*Annoyance*		
Does not feel like this Feels a little bit like this Feels a bit like this Feels quite like this Missing	501 (84.3) 63 (10.6) 23 (3.9) 7 (1.2) 9	0.955 (0.05) 0.874 (0.07) 0.845 (0.08) 0.839 (0.09)
*School work*		
No problems A few problems Some problems Many problems Cannot do Don’t have school children Missing	288 (77.0) 47 (12.6) 17 (4.5) 2 (0.5) 20 (5.0) 180 49	0.956 (0.05) 0.883 (0.06) 0.864 (0.09) 0.915 (0.02) 0.840 (0.06)
*Sleeping*		
No problems A few problems Some problems Many problems Missing	499 (82.9) 78 (13.0) 20 (3.3) 5 (0.9) 1	0.954 (0.05) 0.886 (0.06) 0.851 (0.10) 0.818 (0.06)
*Daily routine*		
No problems A few problems Some problems Many problems Missing	479 (79.6) 107 (17.8) 13 (2.2) 3 (0.5) 1	0.960 (0.05) 0.879 (0.06) 0.789 (0.08) 0.733 (0.09)
*Activities*		
Can join in with any activities Can join in with most activities Can join in with some activities Can join in with a few activities Can join in with no activities Missing	448 (74.5) 122 (20.3) 19 (3.2) 10 (1.7) 2 (0.3) 2	0.962 (0.05) 0.884 (0.07) 0.851 (0.10) 0.897 (0.06) 0.840 (0.07)

In general, mothers who were more religious were associated with fewer issues in their children’s mental health (Table 4). For example, the children reported reduced worries if their mothers participated in more ORAs (AOR = 0.36; 95% CI: 0.14–0.92; *p* = 0.031) or NORAs (AOR = 0.09; 95% CI: 0.01–0.74; *p* = 0.025). Regarding IRA, the children were associated with fewer school work problems if their mothers experienced the presence of the Divine (AOR = 0.76; 95% CI: 0.61–0.94; *p* = 0.012), agreed that their religious beliefs were behind their whole approach to life (AOR = 0.70; 95% CI: 0.56–0.88; *p* = 0.002), or tried hard to carry their religion over into all other dealings in life (AOR = 0.77; 95% CI: 0.61–0.96; *p* = 0.020).

**Table 4:** Association between maternal religiosity and children’s health-related quality of life (CHU9D) components

	Adjusted OR (95% CI)								
	Worries	Sadness	Pain	Tiredness	Annoyance	School work	Sleeping	Daily routine	Physical activity
ORA	**0.36** **(0.14, 0.92)**	1.37 (0.70, 2.69)	1.07 (0.65, 1.77)	1.00 (0.79, 1.27)	1.22 (0.84, 1.77)	0.81 (0.62, 1.05)	0.79 (0.54, 1.17)	1.42 (0.92, 2.19)	1.06 (0.80, 1.41)
NORA	**0.09** **(0.01, 0.74)**	1.64 (0.76, 3.53)	0.96 (0.52, 1.77)	1.05 (0.80, 1.38)	1.12 (0.73, 1.72)	0.74 (0.52, 1.06)	0.84 (0.53, 1.33)	1.58 (0.96, 2.60)	0.99 (0.71, 1.38)
IR1 (Divine presence)	0.67 (0.34, 1.33)	0.71 (0.38, 1.32)	0.90 (0.57, 1.41)	1.18 (0.95, 1.46)	1.17 (0.84, 1.64)	**0.76** **(0.61, 0.94)**	0.99 (0.72, 1.37)	1.27 (0.86, 1.88)	0.93 (0.72, 1.21)
IR2 (Approach to life)	0.78 (0.39, 1.55)	0.75 (0.40, 1.41)	0.95 (0.60, 1.49)	1.01 (0.81, 1.25)	1.03 (0.74, 1.46)	**0.70** **(0.56, 0.88)**	1.21 (0.87, 1.67)	1.24 (0.84, 1.85)	0.94 (0.72, 1.22)
IR3 (Carry over into life)	0.83 (0.42, 1.64)	0.79 (0.42, 1.50)	0.99 (0.63, 1.57)	1.03 (0.83, 1.28)	1.04 (0.74, 1.47)	**0.77** **(0.61, 0.96)**	1.06 (0.76, 1.48)	1.30 (0.87, 1.94)	1.03 (0.80, 1.34)

Maternal religiosity was measured using the five dimensions of the DUREL—OR: organised religious activity (e.g., religious service); NOR: non-organised religious activity (e.g., prayer/meditation), IR1: experienced the presence of the Divine; IR2: religion as underlying approach to life; IR3: carrying religion into other life dealings. Health-related quality of life was measured with the CHU9D

### Children’s Lifestyles

Table 5 shows that the influence of mothers’ religiosity characteristics on their children’s lifestyle was minor. But the children of mothers who participated in ORAs less frequently spent significantly less time playing console games on weekdays (mean 0.6 h, *p* = 0.017). In contrast, the children of mothers who participated in NORAs less frequently spent significantly more time using internet or computers on weekdays (mean 2.6 h, *p* = 0.001) and playing sports at school on weekdays (mean 2.5 h, *p* = 0.004).

**Table 5:** Relationship of religious components on children lifestyle

	Watching TV/DVD		Using internet or computer		Playing game console		Leisure time activities		Sports outside of school		Sports at school
	Mon–Fri	Sat–Sun	Mon–Fri	Sat–Sun	Mon–Fri	Sat–Sun	Mon–Fri	Sat–Sun	Mon–Fri	Sat–Sun	Mon–Fri
*ORA*											
> 1 per month ≤ 1 per month Never	8.2 7.4 8.5	5.2 5.4 5.5	1.6 1.4 1.2	0.9 1.0 0.9	**1.5** **0.6** **1.2**	1.2 0.9 1.4	6.3 6.8 6.1	4.2 5.1 4.9	1.0 1.0 0.9	0.4 0.7 0.5	1.3 1.6 1.1
*NORA*											
> 1 per week ≤ 1 per week Rarely/Never	8.3 7.9 8.3	4.9 5.3 5.6	**1.3** **2.6** **1.1**	0.8 1.5 0.9	0.9 1.3 1.2	0.9 1.4 1.3	6.8 5.7 6.3	4.8 3.5 4.9	0.9 0.9 1.0	0.6 0.6 0.5	**1.4** **2.5** **1.1**
*IR1*											
True Unsure/Not true	8.4 8.2	5.4 5.5	**1.7** **1.1**	1.1 0.8	1.0 1.2	1.2 1.3	6.5 6.2	4.8 4.8	1.1 0.9	0.7 0.5	1.4 1.1
*IR2*											
True Unsure/Not true	7.9 8.3	5.2 5.6	**1.8** **1.1**	1.1 0.9	1.0 1.2	**1.0** **1.4**	6.5 6.2	4.6 4.9	1.0 0.9	0.7 0.5	1.6 1.1
*IR3*											
True Unsure/Not true	8.8 8.1	4.9 5.6	1.5 1.2	0.9 1.0	1.1 1.2	1.0 1.3	6.5 6.3	4.6 4.9	1.0 0.9	0.6 0.5	1.7 1.1

Data are mean hours

Bolded data indicate significant difference between groups of mothers with different levels of religiosity involvement or belief

Intrinsic religiosity did not significantly affect children’s lifestyles. But mothers who experienced the presence of the Divine were associated with longer internet or computer time for their children on weekdays (mean 1.7 h, *p* = 0.033). Mothers whose religious beliefs lie behind their whole approach to life were associated with longer internet or computer time for their children on weekdays (mean 1.8 h, *p* = 0.035) but less time playing game consoles on weekends (mean 1.0 h, *p* = 0.049).

## Discussion

Religiosity is an important factor in women’s health-related attitudes and decision-making (Arousell & Carlbom, 2016; Seybold & Hill, 2001) and has a marked downstream impact on their children’s HRQOL and lifestyles. This study contributes to growing evidence concerning the nature of religiosity in mothers in Australia. In particular, the five dimensions of maternal religiosity (ORA, NORA and three forms of IR) are related to the women’s education level, employment, smoking, living area satisfaction, household income, number of children ≤ 16 years in the household, household members’ alcohol consumption, and decision-making, but to different extents.

### Maternal Characteristics Most Associated with Religiosity

Across all five forms of religiosity, the women more likely to engage in religious activities had higher education levels, included religious values in decision-making, and lived in households with fewer binge drinking problems. While religious women appear more likely to have lower living area satisfaction, most of the participants reported feeling safe in their community and experienced only low psychological distress.

A notable difference is observed between different religious practices in household income: those with higher income are more likely to engage in NORAs than organisational or inherent practices. While a higher income may be the result of paid work, it was found that mothers unemployed were on average three times more likely to engage in ORAs. While initially perplexing, given the general financial circumstances of Australian students (Bexley et al., 2013), this is supported by the fact that higher education levels are also linked to increased religiosity in all forms of religion (see Table 2).

Further, since most participants were married or living with a partner and the partner’s contribution to the household income were not examined in greater detail, some participants may report high household income without being employed themselves. Finally, since greater educational attainment generally supports higher income potential (Blaug, 1947; OECD, 2019), it is simultaneously notable and unsurprising that both higher income and education should be positively associated with religion.

### Religiosity and Stability of the Family Environment

While women who had lower living area satisfaction were more likely to engage in IRAs, most participants reported low levels of psychological distress and felt safe in the community. The majority ate dinner as a family at a table at least once a week and although most did not have private health insurance, only a quarter reported financial hardship.

Intriguingly, lower living area satisfaction is more likely when mothers practice intrinsic religion, rather than attending ORAs or NORAs. This could be due to the incorporation of religious principles in everyday life, which may affect the sense of local community more regularly than organisational religious meetings or private practices. A range of household factors, particularly binge drinking by household members, were associated with all forms of religiosity, while the number of children ≤ 16 years in the household was associated with IRAs only.

Although it is generally thought that greater religiosity supports the formation of stronger familial bonds (Mahoney, 2010), this may not be the case in all situations; indeed, greater religiosity may inhibit the resolution of domestic issues (Dollahite et al., 2018). Thus, religious practises may play both positive and negative roles in the family environment that are less likely to be captured by global assessment tools. In studying the connection between religion and family dynamics, Mahoney (2010) noted the importance of ‘higher resolution’ assessment tools capable of exploring the role of religiosity in its many contexts without losing sight of the underlying reasons for the associations observed.

### Effect of Maternal Religiosity on Children’s Quality of Life and Lifestyles

The CHU9D results show that the mothers’ religiosity, particularly in the IR dimensions, positively influenced their children’s HRQOL regarding schoolwork difficulties. Children’s HRQOL is affected by intrinsic religiosity and is higher when their mothers experience the presence of the Divine and see religion as underlying their approach to life. Their children, however, used more internet/computers during the week but had less time playing games on weekends. Further, the ORA and NORA dimensions positively influence children’s HRQOL regarding worries.

The children of mothers participating in ORAs more frequently spent longer playing games on weekdays. In contrast, those whose mothers engaged in NORAs spent less time on internet/computers and school sports during the week. This contrast could be the result of factors dependent on each family’s values and preferences, and whether children joined their mothers in attending religious services (and thus, simply had less time available). The effect on children’s lifestyles was less pronounced for carrying intrinsic religiosity over into all dealings in life. Better HRQOL is associated with a balance between physical activity and screen time (Lacy et al., 2012; Motamed-Gorji et al., 2019); during early childhood, HRQOL is higher in children who are more active and have less screen time (Del Pozo-Cruz et al., 2019), suggesting that those children who spend less time engaging with this technology may have higher quality of life.

### Establishment and Potential Benefits of Religiosity

The findings help to target health interventions in religious community contexts regarding factors involving education, employment, smoking, alcohol consumption, income, and the family/household characteristics of mothers that directly related to their religiosity and consequently influenced their children. This influence prompts the question of how religiosity is established in families, particularly where religious mothers are involved in making important decisions.

The results in Tables 4 and 5 show that maternal religiosity, especially the intrinsic dimensions, can influence children’s worries, schoolwork, and leisure hours (internet/computers, game consoles, sports). Further, religiosity offers resources that can mediate risk behaviours and promote positive outcomes. Recent studies found that religiosity in adolescents and emerging adults was protective against early onset substance use and later development of abuse problems (Kiesner et al., 2010; Pitel et al., 2012; Porche et al., 2015), as well as self-injurious thoughts and behaviours (Amit et al., 2014). Thus, parental religiosity can play an important role to establish routine religious activities with their children in early childhood.

As women frequently increase their religious participation after becoming mothers (Uecker et al., 2016) and parental influence becomes more distal compared to that of peers from adolescence into early adulthood (Porche et al., 2015), establishment of religiosity in children during early childhood is critical. Overall, the effects of the sociodemographic variables on religiosity considered here are significant but varied, and an understanding of their role may guide policymakers in planning health interventions in religious communities.

### Strengths and Limitations

The key strength of this study was its population-based sample and the inclusion of a wide variety of domains measured at baseline and the 5-year follow-up, which increases the generalisability of the findings. However, there is potential underrepresentation of relatively disadvantaged mothers who were excluded from the recruitment of the study (e.g., those at risk for childbirth complications (Ng et al., 2014) or who did not return the 5-year follow-up questionnaires; e.g., those with lower education levels (Ng et al., 2016)). Therefore, the findings should be interpreted with this in mind. Moreover, as with most cohort studies, a degree of measurement error could be expected to arise from the self-administered nature of the survey data collection. Such errors could affect the accuracy of the self-reported exposure and outcome variables considered in this study.

Although the DUREL was designed for Western religions (e.g., Christianity), it may be adapted to assess religiosity in Eastern religions (e.g., Buddhism) through minor rewording (e.g., exchanging ‘church’ for ‘temple’) (Koenig & Büssing, 2010). However, this was not done in the present study, as no specific religion was under investigation and rewording to cover all possible religions would be clumsy and potentially confusing for participants. The different faiths of the participants were not considered in this work. Nevertheless, given that some religions differ substantially in, for example, their economic attitudes and gender role ideals (Inglehart & Norris, 2003), future studies might examine whether there are corresponding variations in their associations with decision-making, health-related outcomes and children’s quality of life.

## Conclusion

The five forms of maternal religiosity are most closely associated with higher education levels and abstinence from binge drinking in the household. Beyond its role in decision-making, a mother’s religiosity affects her children’s HRQOL. Mothers who were more religious were associated with fewer issues in their children’s mental health and quality of life. The children of mothers who participated in fewer religious activities reported a significantly increased number of worries and problems in their schoolwork. Religiosity also affects children’s lifestyles: those whose mothers participated in NORAs, or IRAs frequently spent more time engaging with technology through internet/computers.
